# The effect of resistance training on PCSK9 levels in patients undergoing cardiac rehabilitation after coronary artery bypass grafting: a randomized study

**DOI:** 10.1186/s12872-023-03571-7

**Published:** 2023-11-09

**Authors:** Bambang Dwiputra, Anwar Santoso, Budhi Setianto Purwowiyoto, Basuni Radi, Ade Meidian Ambari

**Affiliations:** Department of Cardiology and Vascular Medicine, Faculty of Medicine, Universitas Indonesia/ Harapan Kita National Cardiovascular Center, Jl Letjen S Parman Kav 87, Palmerah, Jakarta Barat, 11420 Indonesia

**Keywords:** Coronary artery bypass, PCSK9, Resistance training, LDL cholesterol, Cardiac rehabilitation, Translational medicine

## Abstract

**Background:**

Resistance training is commonly recommended as part of secondary prevention for post-coronary artery bypass graft (CABG) patients in conjunction with aerobic exercise. Despite its potential benefits, there is currently a lack of studies investigating the impact of resistance training on proprotein convertase subtilisin kexin 9 (PCSK9).

**Aim:**

To evaluate the effect of intensive resistance training on proprotein convertase subtilisin kexin 9 (PCSK9) levels among post-CABG patients undergoing cardiac rehabilitation (CR).

**Methods:**

In this prospective, open-label, randomized trial, 87 post-coronary artery bypass graft (CABG) patients were randomly assigned into two groups: moderate to high intensity resistance training and aerobic training (*n* = 44) or aerobic training alone (*n* = 43) for a total of 12 sessions. Changes in PCSK9 levels was determined as a primary endpoint, while secondary endpoints included changes in the six-minute walk test (6-MWT) results, aerobic capacity, WHO-5 well-being index, fasting blood glucose, and lipid profile. Both groups underwent intention-to-treat analysis.

**Results:**

Following completion of cardiac rehabilitation program, the intervention group demonstrated a significant decrease in mean PCSK9 levels when compared to the control group (β = -55 ng/ml, 95% CI -6.7 to -103.3, *p* = 0.026), as well as significant improvements in the 6-MWT result (β = 28.2 m, 95% CI 2.4–53.9, *p* = 0.033), aerobic capacity (β = 0.9 Mets, 95% CI 0.1–1.7, *p* = 0.021), and WHO-5 well-being index (β = 8.1, 95% CI 2.0–14.4, *p* = 0.011) in patients who received resistance and aerobic training. No statistically significant changes were observed in fasting blood glucose, cholesterol, LDL-C, HDL-C, and triglyceride levels.

**Conclusion:**

Resistance training in CR significantly reduced PCSK­9 levels and increases patient’s functional capacity and quality of life. (NCT02674659 04/02/2016).

## What is already known

- PCSK9 is an enzyme which plays a vital role in regulation of cholesterol homeostasis, and reduction in PCSK9 is associated with lower LDL level and subsequently lower cardiovascular disease risk
- Previous research has shown that physical activity could reduce PCSK9 level in healthy participants
- Resistance training is associated with lower systolic blood pressure (SBP), diastolic blood pressure (DBP), and better lipid profile in healthy participants

## What this study adds

- In post-CABG patients, resistance training could decrease PCSK9 levels, improve quality of life and functional capacity significantly compared to controlled intervention.

## How this study might affect research, practice, and policy

- Resistance training provides added benefit for post-CABG patients through reduction of PCSK9 levels, which will potentially improve LDL profiles in these patients.

## Introduction

Cardiac rehabilitation (CR) has been established as an integral part of management in secondary prevention for patients after coronary artery by-pass graft (CABG) surgery [1]. A recent meta-analysis reported that exercise-based CR has been shown to increase physical performance after myocardial revascularization [2]. Exercise training has also been shown to enhance circulatory function and improve oxidative capacity of the muscles in patients with heart disease [3].

A reduction in low density lipoprotein (LDL) cholesterol is also considered as one of the target achievements in secondary preventive strategies in CVD. In the last decade, plasma LDL has been well-associated with Proprotein Convertase Subtilisin Kexin Type 9 (PCSK9) level. PCSK9 is an enzyme produced by hepatocyte, renal mesenchyme, small intestine, and colon epithelial cells. It plays a vital role in the regulation of cholesterol homeostasis by binding to epidermal growth factor-like repeat-A domain of LDL receptor, which targets it for lysosomal destruction. Therefore, a low level of PCSK9 correlates with reduced LDL level. Although the use of statin has been widely recommended to manage dyslipidemia, statin resistance has become clinically relevant. This phenomenon might be attributed to statin-induced upregulation of PCSK9 levels [4]. Based on these updates, Kamani et al. demonstrated that daily physical activity at workplace resulted in lower LDL and PCSK9 level in healthy individuals [5]. Boyer et al. revealed that 1-year lifestyle modification consisting of 150 min/week of physical activity and diet improvement can reduce PCSK9 levels in post-CABG patients [6]. Moreover, Makela et al*.* showed that middle-aged patients (mean age 58 years old) who undergone 3-month physical activity program consisting of 60 min sessions of three times a week have lower PCSK9 levels [7].

However, the intensity of exercise training that offers the most optimal benefit on the lipid profile remains questionable [8]. Aerobic training (AT) and resistance training (RT) appear to offer similar benefits on bone mineral density, glucose tolerance, and insulin sensitivity [9]. American Heart Association (AHA) recognizes resistance training as one of the exercise modalities that produces superior advantage in term of the development of muscle strength, endurance and mass, which facilitates in expending calories via increased maintenance of basal metabolic rate, promoting independence, and preventing falls in the older, frail cardiac patients [10]. These are fundamental to achievement of general health, optimal functional capacity and quality of life (QOL) [11]. American College of Sport Medicine recommends a combination of aerobic and resistance training to enhance health components. This is corroborated by a meta-analysis performed in middle-aged or elderly patients with CAD. A combination of AT and RT versus AT or RT alone versus usual care did improve muscle strength, peak maximal oxygen consumption and mobility significantly in these patients. Furthermore, partial substitution of AT with RT in male with borderline high blood LDL cholesterol was found to produce comparable significant reduction in LDL compared to full duration of AT alone. This may mean that RT could successfully compensate for reduction in aerobic exercise, conferring synergistic physiological benefit and making it potentially more effective [12]. A clinical paradigm is also currently shifting towards progressive functional activity and a controlled upper body therapeutic exercise with less restrictive sternal precautions following a median sternotomy in CABG patients. The use of RT could facilitate bone healing and remodeling in response to loading, optimizing function recovery, pain reduction and QOL [13].

There is still limited evidence on how RT mediates improvement in LDL levels via reduction of PCSK9 level in post CABG patients. Therefore, this study aims to analyze how resistance training influences PCSK9 level in post CABG patients. This study will add value to the mounting evidence on the possible novel therapeutic effect on PCSK9 in the treatment of hypercholesterolemia, especially in post CABG patients.

## Methods

### Study design

We conducted this randomized, controlled, single-blind clinical trial in the National Cardiovascular Center Harapan Kita (NCCHK) Jakarta, Indonesia, from February to December 2016. Patients who underwent CABG surgery were randomly assigned to outpatient CR programs that consisted of aerobic training and RT component (intervention group) and aerobic training only (control group). This CR program is supervised by cardiologists. Patients who fulfilled the eligibility criteria were consecutively recruited until the required minimum sample size was reached.

The RT consisted of three incremental stages of free weight training that involved upper and lower extremities (12 sessions in total). The study evaluated the change of PCSK9 levels after CR as the primary endpoint. Secondary endpoints included the change of cardiometabolic risk factors (body mass index, systolic and diastolic blood pressure, fasting blood glucose, and lipid profile).

### Eligibility criteria and patient recruitment

In the beginning of the study, post elective CABG surgery patients more than 18 years of age or older who underwent CR program in NCCHK were recruited. The phase II CR is defined as a program conducted in the outpatient setting (center-based) after patient discharge. CABG surgery was performed within a one month period. Patients are eligible to begin an early phase II CR program if their six-minute walking test (6MWT) results > 240 m and Hb > 10 mg/dL. Participants with unstable angina, severe hypertension, acute heart failure, malignant arrhythmia, neuromuscular diseases, obstructive pulmonary diseases, stroke, and musculoskeletal disabilities were excluded from the study. This study also excluded CABG surgery patients with concomitant valve replacement or repair and congenital heart disease procedures.

### Study procedures

#### Study participants

Eligible participants entered a single-blind run-in phase using concealed block randomization, during which they received phase II CR with or without additional RT for 6 weeks. Phase II CR was performed 4 weeks after patients were discharged from hospitals following CABG surgery. Computerized randomization with varying block size was performed by a third-party statistician. The participating cardiologists recruited the study participants following the study protocol.

#### Data measurement and collection

Before and after phase II CR, baseline data were measured such as body weight, body height, lipid profile (total cholesterol, LDL, HDL, triglyceride), fasting blood glucose, PCSK9, and liver function in all subjects. Blood samples were taken after patients fasted for 12 h and measured at the NCCHK laboratory unit; while PCSK9 level examination was assayed at the Division of Research and Development laboratory as follows: peripheral venous blood was taken from the subjects into a tube containing Ethylene diamine tetra-acetic acid (EDTA). The samples were then centrifuged at 1000 rpm for 15 min to isolate the plasma. Enzyme-linked Immunosorbent Assay (ELISA) was used to measure PCSK9 (R&DSystems® catalog number DPC900, Minneapolis). The result was then read at 450 nm wavelength. All clinical data related to CABG surgery were derived from medical records. At the end of the study period, the same measurements were taken. In addition, we utilized the WHO-5 Wellbeing Index to measure patients’ quality of life [14].

#### Intervention

The intervention group performed a combination of RT and AT, meanwhile the control group only performed aerobic training. The supervised RT consisted of biceps and quadriceps training. Blood pressure, anthropometric data, baseline electrocardiography (ECG), pain and Borg scales were measured before an exercise program was carried out. The additional RT was composed of 12 sessions performed within 6 weeks (2–3 times per week). Each session is divided into three incremental phases as follows: 1) Phase I (4 sessions): 1 lb./extremity; 2 types of exercise, each 10 repetitions, 2 s concentric/2 s eccentric; 2) Phase II (4 sessions): 2 lb./extremity; 2 types of exercise, each 10 repetitions, 2 s concentric/2 s eccentric; 3) Phase III (4 sessions): 3 lb./extremity; 2 types of exercise, each 10 repetitions, 2 s concentric/2 s eccentric. Exercise dosage was increased by weight increment to ensure progressive overload as individuals progressed, hence facilitating improvements in muscular strength and endurance [10]. These weights correspond to a perceived exertion during RT, approximating 11 to 14 on the Borg scale in most participating individuals. Because this was a pilot study in our population of patients following CABG surgery, we followed the conventional guidelines to impose a somewhat restrictive weight limit for the first 4 weeks after a cardiac intervention to maximize the safety of our intervention. Patients received clear instruction on proper breathing to prevent Valsava’s response [10]. Any adverse signs and symptoms, for example dizziness, dyspnea, chest pain, or heart rhythm abnormalities were monitored and RT was discontinued immediately should it occur.

Aerobic training protocol was individualized based on six-minute walk test (6MWT), following the protocol developed by Cardiac Prevention and Rehabilitation Division in NCCHK that adopted guideline from American College of Sport Medicine [15]. In 6MWT, patients were instructed to walk as quickly as possible within 6 min over a 45-m walkway in the gymnasium in NCCHK, in accordance with the American Thoracic Society guideline [16]. Patients were permitted to slow down, to stop or to rest as necessary, but would resume walking as soon as they were able to. Heart rate, blood pressure, and oxygen saturation were measured before and after the test.

At the end of study, an exercise stress test was performed with Bruce protocol (GE Healthcare, USA) without a gas analyzer. Peak metabolic equivalent, [17] was estimated based on the participants’ grade and speed attained on the treadmill at the maximum exercise tolerance.

### Statistical analysis

A sample size of 42 patients was calculated with a power calculation of 0.8 to detect at least 80 ng/mL of PCSK9 level for meaningful clinical effect [5]. To test for differences in demographics between groups, unpaired Student’s t-test (for continuous variables) and chi-square test (for categorical variables) was performed. Next, univariate linear regression was used to assess the effect of RT on the study outcomes. Missing values of primary outcomes were not imputed for statistical analysis (per protocol analysis). Since the number of dropout rates was few and reasons were similar in both groups, we assumed per protcol analysis would still maintain the prognostic balance of randomization in the beginning of the study. The independent variable was the exercise group (control or intervention), and the dependent variable was the study outcomes. The effect size was expressed in the regression coefficient (β) with the confidence interval (CI) and *p*-value. A value of *p* < 0.05 corresponding with CI that did not cross zero was considered statistically significant. Patients and the public were not involved in the design, conduct, reporting, or dissemination of this research.

## Results

**Fig. 1:**
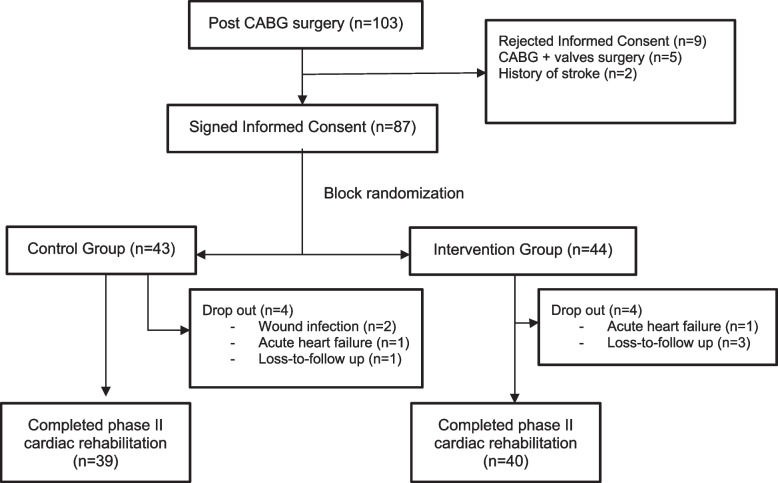
Study protocol

Eighty-seven patients who fulfilled the eligibility criteria had signed an informed, written consent form according to the Declaration of Helsinki. After block randomization, 44 patients were assigned to the intervention group, and 43 patients were assigned to the control group. Four patients from each group were dropped out of the study for the following reasons: 1) Two patients (2.3%) had acute decompensated heart failure during the phase II CR, 2) Two patients (2.3%) needed medical treatment caused by surgical site infection, 3) Four patients (4.6%) were lost to follow-up due to transportation issues. Therefore, exercise training was discontinued (Fig. 1).

The population characteristics were recorded as mean and standard deviation for numerical data and percentage for categorical data (Table 1). The mean age of the patients in both groups was similar (57.2 (SD: 8.4) vs. 56.3 (SD: 8.1) years old) with the majority of subjects being male (88.4% vs. 88.6%). The mean ejection fraction for control group vs intervention group were (52.1 (SD: 15.2) vs. 50.2 (SD: 15.4). Baseline characteristics were similar in both groups.

**Table 1:** The characteristics of the study population

	**Control (*n* = 43)**	**Intervention (*n* = 44)**
Age (year old)	57.2 (8.4)	56.3 (8.1)
Men, n (%)	38 (88.4)	39 (88.6)
Height (cm)	164.5 (6.8)	162.9 (6.4)
Weight before exercise (33)	68.2 (10.6)	65.9 (10.9)
Waist circumference (cm)	97.5 (10.5)	95.1 (10.8)
Body mass index before exercise (kg/m^2^)	25.2 (2.9)	24.6 (3.1)
Coronary heart disease (CHD) risk factors		
Hypertension (n,%)	32 (74.4)	31 (70.5)
Diabetes Mellitus (n,%)	20 (46.5)	21 (47.7)
Dyslipidemia (n,%)	21 (48.8)	21 (47.7)
Smoking (n,%)	32 (74.4)	33 (75)
The family history of CHD (n,%)	6 (14)	8 (18.2)
Duration of hospitalization (days)	9.1 (4.0)	9.1 (3.9)
Duration of CPB (minutes)^b^	81.5 (26.4)	81.2 (21.3)
Duration of aortic cross-clamp (minutes)^b^	48.9 (24.1)	47.6 (16.9)
Duration of exercise (days)	22.7 (7.4)	22.6 (8.1)
Frequency of exercise		
3x/week (n,%)	18 (41.9)	20 (45.5)
5x/ week (n,%)	25 (58.1)	24 (54.5)
Ejection fraction (%)	52.1 (15.2)	50.2 (15.4)
Systolic BP before exercise (mmHg)	121.1 (14.8)	125.3 (17.9)
Diastolic BP before exercise (mmHg)	74.5 (9.8)	76.8 (9.9)
Fasting blood glucose before exercise (mg/dl)	116.5 (41.5)	116.6 (37.6)
Lipid profile before exercise		
Total cholesterol (mg/dl)	161.1 (32.4)	160.4 (36.5)
High density lipoprotein (mg/dl)	32.9 (8.5)	34.5 (8.0)
Low density lipoprotein (mg/dl)	96.7 (30.1)	96.3 (30.2)
Triglyceride (mg/dl)	172.2 (63.2)	171.8 (58.7)
SGOT before exercise (mg/dl)	24.6 (16.5)	28.8 (16.6)
SGPT before exercise (mg/dl)	44.3 (23.3)	49.2 (24.2)
Uric acid (mg/dl)	7.1 (1.1)	7.1 (1.6)
Creatinine (mg/dl)	1.07 (0.3)	1.03 (0.2)
Statin therapy		
Simvastatin, (n, %)	32 (74.4)	33 (75.0)
10 mg (n, %)	4 (9.3)	3 (6.8)
20 mg (n, %)	28 (65.1)	30 (68.2)
Atorvastatin 20 mg, (n, %)	11 (25.6)	11 (25.0)
PCSK9 before exercise (ng/ml)^a^	382.4 (127.3)	407.04 (112.3)

Data were recorded in mean (SD) unless otherwise stated

^a^n control = 39, n intervention = 40

^b^n control = 38, n intervention = 40

**Fig. 2:**
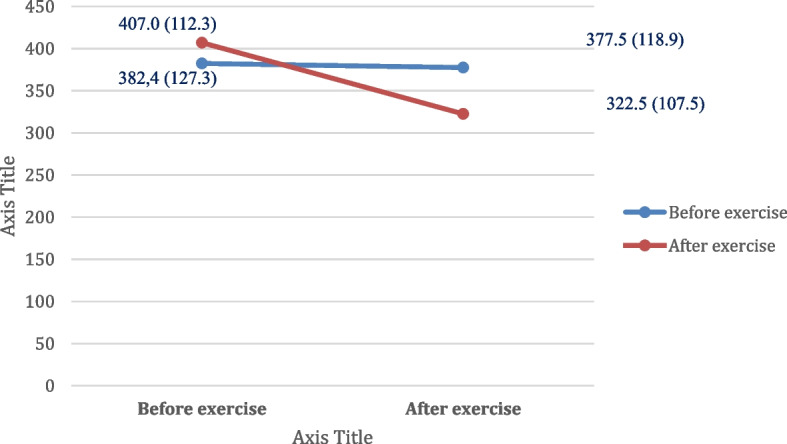
The comparison of the PCSK9 level before and after exercise intervention

The result of PCSK9 levels in patients after phase II CR is shown in Table 2. There was a significant difference in PCSK9 levels between the control and the intervention group. After undergoing phase II CR, the group who received RT showed a more significant decrease in PCSK9 levels compared to the control group. Resistance exercise training in phase II CR had reduced PCSK9 level of 55,0 ng/ml compared to the control group (CI 95% -6,7 (-103,3)), *p* = 0.026). The comparison between the PCSK9 levels before and after exercise is shown in Fig. 2.

**Table 2:** The effect of resistance exercise training on PCSK9 level

	**Control (*n* = 39)**	**Intervention (*n* = 40)**	**Regression coefficient (β)**	**CI 95% min(max)**	***p* value**
PCSK9 (ng/ml)	377.5 (118.9)	322.53 (107.5)	-60.5	-7.5 (-113.4)	0.026^*^

Data were recorded in mean (SD)

^*^p < 0.05

The anthropometric measurement among patients after exercise training did not show significant differences in height, weight, and body mass index in both groups. There were also no significant differences in the hemodynamic parameter, blood glucose, and lipid profile in both groups. No significant differences in LDL levels (*p* = 0.07), total cholesterol (*p* = 0.99), HDL levels (*p* = 0.44), and triglyceride (*p* = 0.56) were found in both groups at the end of phase II CR (Table 3).

**Table 3:** The effect of resistance exercise training on cardiovascular risk factors

	**Control (*n* = 39)**	**Intervention (*n* = 40)**	**β**	**CI 95% min (max)**	***p* value**
Weight (33)	68.4 (10.9)	65.9 (10.8)	-2.41	-7.03 (2.21)	0.30
Waist circumference (cm)	97.5 (10.8)	94.8 (11.4)	-2.71	-7.47 (2.04)	0.26
Body mass index (kg/m2)	25.3 (2.9)	24.7 (3.0)	-0.59	-1.86 (0.69)	0.36
Systolic BP (mmHg)	117.3 (11.4)	118.8 (12.5)	1.42	-3.69 (6.54)	0.58
Diastolic BP (mmHg)	73.8 (6.3)	74.8 (10.3)	1.03	-2.64 (4.69)	0.57
Total cholesterol (mg/dl)	162.2 (32.6)	162.1 (35.5)	-0.10	-14.61 (14.4)	0.99
HDL (mg/dl)	36.1 (7.0)	37.4 (7.9)	1.25	-1.95 (4.44)	0.44
LDL (mg/dl)	106.3 (26.6)	96.2 (24.9)	-10.12	-21.11 (0.87)	0.07
Triglyceride (mg/dl)	145.7 (50.2)	139.4 (50.7)	-6.3	-27.8 (15.2)	0.56
Fasting blood glucose (mg/dl)	107.7 (24.6)	102.4 (22.9)	-5.29	-15.42 (4.84)	0.30
6-Minute Walk Test (m)	360.7 (55.1)	388.9 (65.4)	28.2	2.4 (53.9)	0.033^*^
Aerobic capacity (METS)	6.2 (1.8)	7.1 (1.7)	0.9	0.1 (1.7)	0.021^*^
WHO-5 well-being index	79.8 (17.7)	87.9 (10.7)	8.1	2.0 (14.4)	0.011^*^

Data were recorded in mean (SD)

^*^p < 0.05

## Discussion

The primary finding of this study was that the additional resistance exercise training decreased the PCSK9 level significantly in patients undergoing cardiac rehabilitation after CABG surgery. However, the lower level of PCSK9 was not followed by improvement in cardiometabolic parameters such as body mass index, blood glucose, and lipid profile at the end of CR program.

### Relationship between PCSK9 level and resistance training

To the best of our knowledge, this is the first randomized study to investigate the effect of resistance training on PCSK9 levels in post-CABG patients. It also establishes a correlation between resistance exercise training and PCSK9. A previous study only compared PCSK9 levels before and after lifestyle modifications of 150 min/week of physical activity and diet quality improvement, revealing an unexpected 5.2% increase within a year (*p* = 0.05) despite improvements in some lipoprotein profiles. The author couldn't conclude if this increase was biologically linked to cardiometabolic parameters or due to better adherence to lifestyle modification counseling. Notably, the study lacked control groups [6].

Intervention in this study showed significant decrement of PCSK9 level after exercises. Previously, Kamani, et al. [5] concluded that physical activity could decrease PCSK9 level in healthy populations. In individuals using stairs instead of elevators at the office, PCSK9 levels dropped from 403.6 to 324.3 ng/ml (*p* = 0.001), and LDL cholesterol decreased from 3.5 to 3.3 mM (*p* = 0.01). VO2 max increased from 37.0 to 40.4 ml/kg/min at the end of the intervention. PCSK9 levels decreased in the first 3 months but remained stable after 6 months, possibly due to decreased motivation for exercise. In contrast, Makela et al. [7] found no significant correlation between plasma PCSK9 levels and 160 min/week of aerobic physical activity in 68 high-risk type 2 diabetes subjects. Their results may be affected by seasonal changes in daylight duration, which can impact PCSK9 levels, as they follow a diurnal rhythm [18, 19]. Similar to Kamani, et al. [5], our sampling was mostly taken in the morning when plasma PCSK9 levels were relatively stable.

Resistance exercise training may mediate PCSK9 levels via mechanisms involving Annexin A2. Chen, et al. [20] suggested that it increases Annexin A2 by around 2.8-fold, altering the catalytic domain structure of PCSK9, inhibiting its function in LDL receptor degradation. This leads to increased LDL clearance. Study by Kamani, et al. [5] supported this finding, showing a decrease in PCSK9 resulted in lower LDL cholesterol, associated with Annexin A2 levels at the third and sixth month. RT protocols induce Annexin A2 secretion, suppressing PCSK9 expression, potentially explaining the lower LDL in the intervention group post-study.

### Relationship between resistance training and functional capacity

Our study showed improved functional capacity in post-CABG patients after just 4 weeks of resistance training, with higher METS and 6-MWT distance. In chronic heart failure patients, resistance training alone significantly improved 6-MWT compared to the non-training group. The clinical significance of this benefit for chronic heart failure patients remains to be determined. Notably, in CABG patients, a 6-MWT distance of less than 250 m is associated with a higher risk of rehospitalization, with a relative risk of 16.74 (95% CI: 2.53–110.78, *p* = 0.008) [21].

RT enhances maximal workload tolerance through peripheral muscle adaptations like increased cross-sectional area, capillarization, and oxidative capacity [22]. Skeletal muscle myopathy, including impaired metabolic capacity and fiber transformation, can limit exercise capacity [23]. RT reduces resting and submaximal exercise plasma norepinephrine levels, promoting increased blood flow during activities like the 6-MWT [24]. Because RT targets specific muscle groups, it directly influences blood flow and skeletal muscle metabolism, independent of central hemodynamic adjustments. Minimal changes in circulatory dynamics were observed during isotonic exercises with various intensities, indicating that RT is well-tolerated and imposes lower cardiac demands compared to aerobic exercise [25].

Peak VO2 is the gold standard for cardiorespiratory fitness, but it wasn't measured due to equipment limitations on gas analyzer [26]. Furthermore, this technique could not be well tolerated by elderly patients or those with comorbidities [27, 28]. Prior research indicated a strong correlation between peak MET estimated on a treadmill test and the 6-MWT, a reflection of daily activities and exercise capacity [28]. We selected the 6-MWT as it's easy to administer and safer for older patients and those with musculoskeletal conditions like knee arthrosis, minimizing the risk of instability or falls [29].

### Relationship between resistance training and quality of life

In our study, patients showed improved quality of life via the WHO-5 well-being index. Fan, et al.'s meta-analysis suggested that combining RT and AT is more effective in enhancing physical and global QOL scores in CVD patients than AT alone, driven by improved cardiopulmonary function, peak oxygen uptake, muscle strength, and aerobic capacity. A previous study in chronic heart failure patients also explored resistance training alongside aerobic training, showing a favorable but nonsignificant trend in favor of the combination. Peak VO2 is independently associated with QOL and activity capacity [30]. Enhanced muscle strength from training is linked to a better quality of life [31]. Given RT's effectiveness in improving muscle strength, adding it to exercise regimens may further enhance QOL.

### Relationship between resistance training and cardiometabolic risk factors

In our post-CABG patient study, resistance training in phase II CR did not improve cardiometabolic parameters like BMI, blood glucose, and lipid profile. Previous studies, however, have shown positive effects of resistance exercise, such as reduced blood pressure, improved BMI, muscle strength, and lipid profile. Cornelissen, et al. [32] conducted a meta-analysis in healthy adults (> 18 years) and found a significant decrease in blood pressure in normotensive and prehypertensive individuals across 25 studies, with no difference in hypertensive cases. Body fat and triglyceride levels decreased by 0.6% (*p* = 0.01) and 0.11 mmol/L (*p* = 0.05), respectively, while other lipid components and fasting blood glucose showed no significant changes. Additionally, some trials reported reductions in systolic and diastolic blood pressure, triglycerides, LDL cholesterol, CRP levels, VLDL, and blood glucose after using RT as intervention [33–35].

Boyer, et al. [6] studied 1-year lifestyle modification (150 min/week of physical activity and diet quality improvement) in post-CABG patients, resembling our study's population. Both studies featured primarily statin-treated patients with some achieving target LDL levels. The male-only population in their study is on par with our study’s population. In Boyer's study, LDL cholesterol and apoB levels remained unchanged, potentially due to its non-randomized design, limited gender selection, and lack of a control group. The similar patient characteristics might explain why our study participants also didn't show lipid profile improvements in phase II CR.

In our study, patients received a lower statin dose, deviating somewhat from current guidelines due to financial constraints under the National Health Insurance System. Statin doses remained constant to isolate changes in PCSK9 levels resulting from the intervention. Notably, the control group showed a slight increase in LDL cholesterol at the study's end. However, we did not account for patients' dietary habits, which could have impacted the LDL cholesterol level of both the control and the intervention group.

While PCSK9 levels decreased in our phase II rehabilitation study, no significant improvements were observed in cardiovascular risk factors (blood pressure, blood glucose, and lipid profile). This may be due to the initial control of these factors through medication and the study's relatively short duration, potentially insufficient to impact cardiovascular risk factors. In contrast, some studies demonstrated cardiovascular risk factor improvements after six weeks of resistance training and aerobic with resistance training [8].

Our study aimed to enhance post-CABG patient outcomes by incorporating resistance exercise training in phase II rehabilitation, a crucial transitional phase. To assess the enduring impact of resistance exercise training on cardiovascular risk factors, future research with a larger sample size and a longer duration could be conducted in phase III rehabilitation.

### Study strengths and limitations

The main strength of our study is that it is the first randomized clinical trial that investigates PCSK9 level in post CABG patients who performed resistance training. This study also proves that resistance training can be done safely in post-CABG patients. Further studies are required to demonstrate the relationship between resistance exercise training and patients’ anthropometric and cardiometabolic parameters. Additionally, further studies are warranted to explain the mechanism of resistance exercise training in decreasing the PCSK9 level, especially via the proposed mechanism such as Annexin A2, adiponectin, and insulin resistance measured with HOMA-IR.

Several limitations must be acknowledged within this study. Firstly, we were unable to analyze cardiorespiratory fitness due to equipment limitations related to gas analyzers. Moreover, patients in our study received a reduced statin dosage due to restrictions imposed by the National Health Insurance System, potentially influencing the lipid profiles of the subjects. The dietary choices of our patients may have introduced confounding factors into our study. It is worth noting that lipid profiles are influenced by dietary preferences in addition to physical activities and the use of statins. To address these limitations in future research, we recommend mitigating potential statin effects by assessing lipid profiles when they are already stabilized, thereby measuring the true impact of resistance training on PCSK9. Additionally, the dietary habits of the participants deserve attention. Our study's population size is relatively small and exhibits an uneven distribution of male and female subjects. Finally, further investigation is warranted to explore the effects during phase III of cardiac rehabilitation.

## Conclusions

The addition of resistance training to aerobic training components in cardiac rehabilitation after CABG surgery reduced PCSK9 level significantly. Although it is not statistically significant, we predict that this reduced PCSK9 levels may lead to more advanced LDL reduction in a longer period of intervention.

## Data Availability

All data generated or analysed during this study are included in this published article. Additional information will be made available upon reasonable request by the corresponding authors.
